# Urinary neutrophil gelatinase-associated lipocalin as an early marker of acute kidney injury complicating circulatory shock

**DOI:** 10.1186/cc10960

**Published:** 2012-03-20

**Authors:** H Sherif, M Foda, M Shehata, A Ibrahim

**Affiliations:** 1 Faculty of Medicine, Cairo University, Cairo, Egypt

## Introduction

We evaluated the novel urinary neutrophil gelatinase-associated lipocalin (NGAL) as an early biomarker that rapidly releases in acute kidney injury (AKI) complicating circulatory shock.

## Methods

We measured the urinary NGAL level from collected urine in 45 patients with circulatory shock, during the first 6 hours and after 24 hours. Eleven patients responded to fluid infusion ± vasopressors and were considered as a separate control group.

## Results

The estimated urinary NGAL at day 1 and day 2 post circulatory shock could predict AKI presented at days 2 and 3 and days 3 and 4 (*P *< 0.05, *P *< 0.001 and *P *< 0.001, *P *< 0.001) respectively. Apart from all conventional kidney parameters and biomarkers, significant inverse correlations could be detected only between urinary NGAL at days 1 and 2 with the corresponding urine output in the patient group (*r *= -0.51 and -0.64, *P *< 0.05 and *P *< 0.001, respectively). The best cut-off value of urinary NGAL at day 1 was 26 ng/ml, for which sensitivity was 62% and 69% and specificity was 75% and 80% for prediction of AKI presented at days 2 and 3, respectively. While the best cut-off at day 2 was 29 ng/ml, for which sensitivity was 70% and 74% and specificity was 90% and 80% for prediction of AKI presented at days 3 and 4, respectively. Urinary NGAL at day 2 could significantly predict mortality complicating AKI rather than at day 1 (*P *< 0.05).

## Conclusion

Urinary NGAL seems to be a potential early and sensitive biomarker for AKI and a persistently increased level at day 2 can predict mortality following circulatory shock.

